# Exploring unsupervised learning techniques for early detection of myocardial ischemia in type 2 diabetes

**DOI:** 10.3389/fendo.2025.1668516

**Published:** 2025-10-13

**Authors:** Bing Liu, Yan-jie Hou, Ping Wu, Xiao Han, Hao Qi, Xiu-Yun Yang, Zhi-Fang Wu, Si-Jin Li

**Affiliations:** ^1^ Department of Nuclear Medicine, First Hospital of Shanxi Medicinal University, Shanxi Medical University, Taiyuan, Shanxi, China; ^2^ Collaborative Innovation Center for Molecular Imaging of Precision Medicine, Shanxi Medical University, Taiyuan, Shanxi, China; ^3^ Department of Endocrinology, First Hospital of Shanxi Medical University, Taiyuan, Shanxi, China; ^4^ Modern Educational Technology Center, Shandong First Medical University and Shandong Academy of Medical Sciences, Jinan, China

**Keywords:** machine learning, elbow method, silhouette coefficient, myocardial ischemia, diabetes mellitus, single-photon emission computed tomography

## Abstract

**Introduction:**

Myocardial ischemia can result in severe cardiovascular complications. However, the impact of clinical factors on myocardial ischemia in individuals with T2DM remains unclear. we applied a clustering approach to identify the variability in myocardial ischemia evaluated through Single-Photon Emission Computed Tomography.

**Methods:**

Retrospective statistics derived from 637 T2DM patients with myocardial ischemia who participated in SPECT imaging at our hospital between January 2022 and September 2024 were gathered. Ischemia areas, cavity size, wall motion,ventricular contraction, cardiac systolic coordination, End-diastolic Volume, End-systolic Volume; Left ventricular injection fraction were assessed and analyzed. Clustering analysis of medical data in unsupervised learning, involving the elbow method and silhouette coefficient(cluster 1: 262; cluster 2: 375);.

**Results:**

The Healthcare information between two groups differed in multiple respects (1) Cluster 1 had the had the older patient(63.23 ± 12.31), longer average duration of diabetes(10.27 ± 8.77), higher Glycated Hemoglobin(HbA1c) values(7.69 ± 1.76), the higher level of serum creatinine (115.42 ± 106.18µmol/L);and a higher proportion of patients with insulin treatment(40.5%) (2).Cluster 1 had more males(68.8%),higher proportion of patients with smoking history(44.5%), the higher level of Cholesterol(3.96 ± 1.12mmol/L),serum uric acid (406.78 ± 135.24µmol/L),Low-density lipoprotein cholesterol(2.08 ± 0.32mmol/L),and was more prone to statin therapy (6.1%).The SPECT features differed across the various clusters (1):Cluster 1 had higher proportion of Hypokinesis(38.2%),poor ventricular contraction(57.6%),Impaired Cardiac systolic coordination(63.7%),and abnormal LVEF(81.3%) (2).Cluster 2 had a higher proportion of total ischemia(11.5%) and abnormal ESV(52.8%) (3).There was no significant difference in Ischemia areas, Cavity size, Involved segments, and EDV.

**Discussion:**

Although the unsupervised clustering approach revealed differences in various clinical and imaging characteristics, no significant differences were observed in ischemic burden, cavity size, involved segments, or EDV.

## Graphical Abstract

Summary of the study design and key findings.

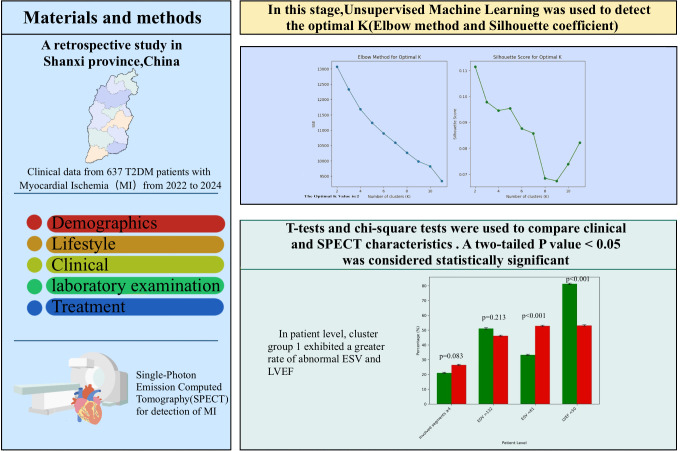

## 1 Introduction

Diabetes is a major global health burden, with its increasing prevalence contributing to higher morbidity and mortality rates (1, 2). Data from the International Diabetes Federation (IDF) indicates that the global population of individuals with diabetes has reached 536 million, and it is expected to rise to 783 million by 2045 (3). The most widespread form of diabetes is T2DM.Studies have indicated that T2DM contributes to a higher risk of cardiovascular diseases (4).

Cardiovascular disease (CVD) is the leading cause of death and disability in T2DM (5, 6). Research has demonstrated that compared to individuals without diabetes, those with T2DM have a two to four times higher risk of cardiovascular disease (CVD) (7, 8). Myocardial ischemia(MI) is a major cardiovascular disease, which is more commonly seen in the diabetic population than in those without diabetes (9, 10). Additional risk factors for cardiovascular death, including hypertension, dyslipidemia, smoking, and visceral obesity are particularly harmful in individuals with diabetes (3, 11). T2DM patients have different clinical features, and the comprehensive effect of these features on myocardial ischemia still needs further study.

Machine learning (ML), a branch of artificial intelligence that enables mining the relationships from complex datasets, has been used to make predictions about future outcomes (12). ML-based techniques have been successfully applied on various types of Coronary artery disease (CAD) datasets (13–19). These algorithms have demonstrated promising performance in the detection and treatment of myocardial ischemia. However, Limited research has been dedicated to exploring the use of non-supervised learning algorithms to differentiate the varied medical features of T2DM and examine the connections across various categories and myocardial ischemia characteristics. Thus, we applied non-supervised learning algorithms to examine The diversity in clinical manifestations of T2DM based on clinical indicators and to better understand the overall impact of medical determinants on myocardial ischemia characteristics detected on SPECT, which could potentially enhance personalized medical intervention.

## 2 Methods

The Biomedical Research Ethics Committee of our hospital approved this retrospective study, and the requirement for written informed consent was waived.

### 2.1 Study cohort

Between January 2022 to September 2024, We retrospectively reviewed T2DM patients with myocardial ischemia identified through SPECT at our hospital. The criteria for exclusion were listed below: Patients previously treated with percutaneous coronary intervention, coronary bypass surgery, and cerebrovascular diseases before SPECT, SPECT image quality was inadequate for ischemia diagnosis; deficient clinical data; Patients with heart disease, respiratory failure, severe liver and kidney diseases, cancer, severe infections, and other illnesses. Finally, 637 patients with T2DM were incorporated into the study.

### 2.2 Acquisition of resting gated MPI

All patients underwent gated resting SPECT MPI using a single IQ-SPECT dual-probe scanner (Symbia T16, Siemens, Germany). After fasting for at least 4 hours, 99mTc-MIBI (740–925 MBq) was intravenously injected. Fatty meals were consumed 15 to 20 minutes prior to imaging. Electrodes were placed on the chest to capture gated data. Patients remained supine with arms raised, and acquisition began 60 minutes post-injection, lasting 8 minutes. Imaging parameters included a 128×128 matrix, 208° rotation, 34 frames, and 25 seconds per frame. Images were reconstructed using ordered subsets expectation maximization in fully automated mode.

### 2.3 SPECT analysis

**Figure 1:**
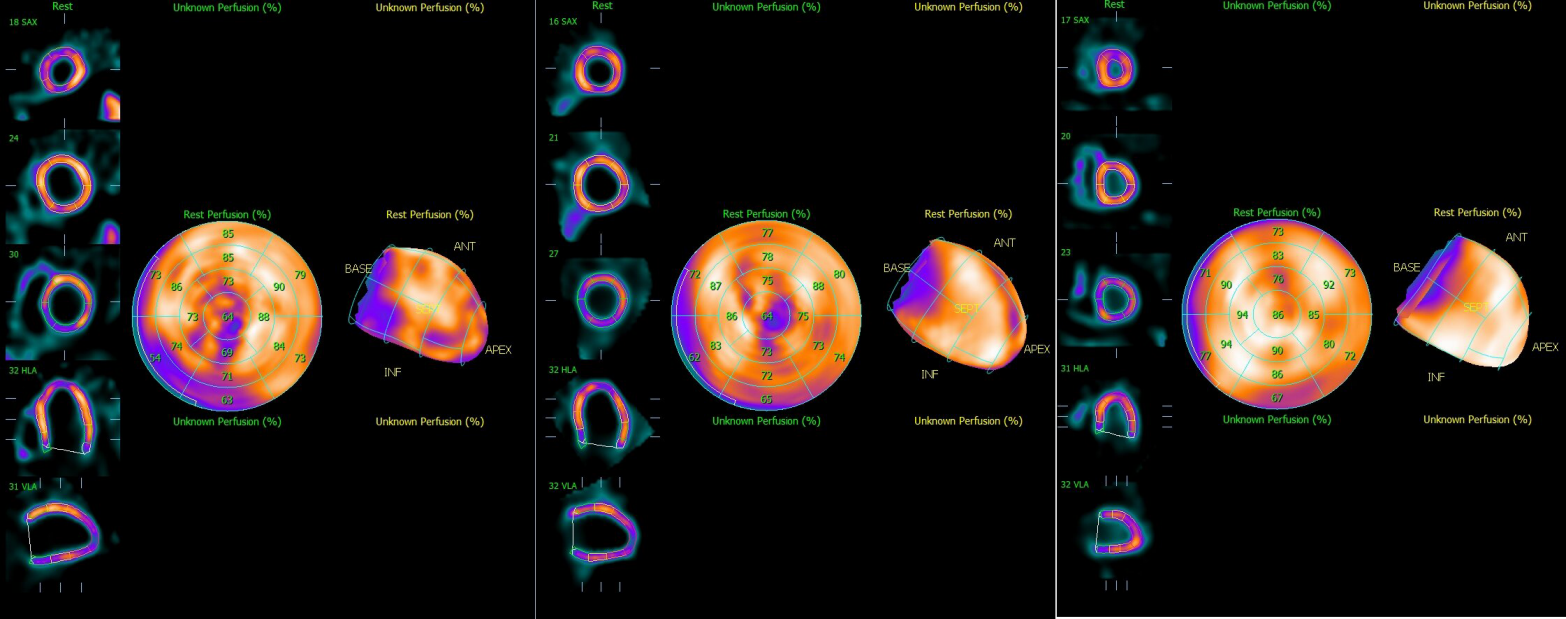
Representative Single-Photon Emission computed tomography images of **(A)** Total ischemia, **(B)** Part ischemia, and **(C)** Normal.

The SPECT and gated SPECT images were anonymized and visually analyzed by two experienced observers. Myocardial perfusion was assessed by consensus using a 17-segment division of the left ventricle (20) and a four-point grading system: 0, normal uptake; 1, equivocal; 2, moderate reduction; and 3, severe reduction. Segments with an uptake score ≥2 at stress were classified as having a definite uptake reduction (21–23). Only segments with an uptake score ≥2 at stress were considered to have a definite uptake reduction at stress. Segments with an uptake score ≥2 at stress were classified as having a definite uptake reduction. Total myocardial counts were determined in each acquisition with a manually adjusted elliptical region of interest (**Figure 1**).

### 2.4 Unsupervised machine learning

**Figure 2:**
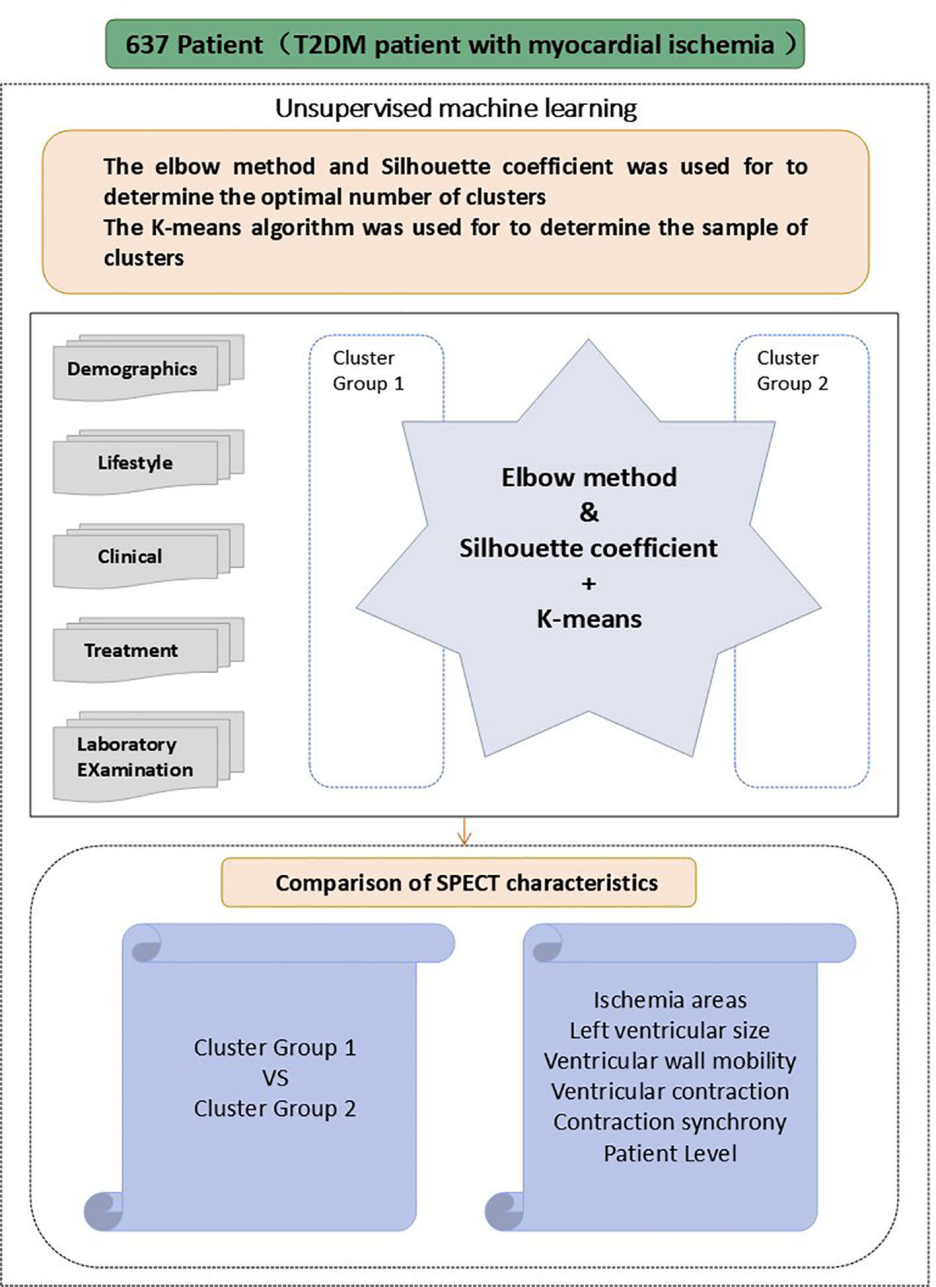
Schematic for the main steps of this study.

The elbow method combined with the Silhouette coefficient were utilized to cluster the 637 T2DM patients based on their clinical characteristics (**Figure 2**), to identify the appropriate cluster count. The core principle of this method is to reduce to the smallest possible value the sum of squared errors between the cluster center and the points within each cluster. As K grows, the model’s separation becomes more distinct. However, when K exceeds the best appraisal, The cumulative squared errors no longer shows significant changes. K-means is a clustering technique used for grouping objects that consist of both quantitative and qualitative data (24).

The fundamental procedures of the Silhouette coefficient and the elbow method are described below:

(1) Select a Range of K values.

Determine a possible range for K, typically starting from 2(since the Silhouette coefficient is meaningless for K = 1)up to a reasonable maximum value.

(2) Perform the K-means clustering.

For each K value, run the K-means algorithm and record the following results:

SSE(Sum of squared Errors):Used for the Elbow Method.

Silhouette Coefficient: Used to evaluated clustering quality.

(3) Analyze the Elbow Method: Calculate SSE, Plot the Elbow Point, Identify the Elbow point.

(4) Analyze the Silhouette Coefficient: Calculate the Silhouette Coefficient, Plot the Silhouette curve, Select The Optimal K Value.

(5) Compare the Elbow Method and Silhouette Coefficient.

To determine the optimal number of clusters, we evaluated both the elbow method and the silhouette coefficient across a range of K values (K = 2–6). The elbow plot showed that the reduction in the sum of squared errors plateaued after K = 2, while the silhouette coefficient reached its maximum at K = 2. These complementary results indicated that two clusters provided the best balance between separation and stability. Moreover, the two-cluster solution yielded clinically meaningful subgroups with distinct demographic, biochemical, and imaging characteristics, further validating the selection of K = 2.

**Figure 3:**
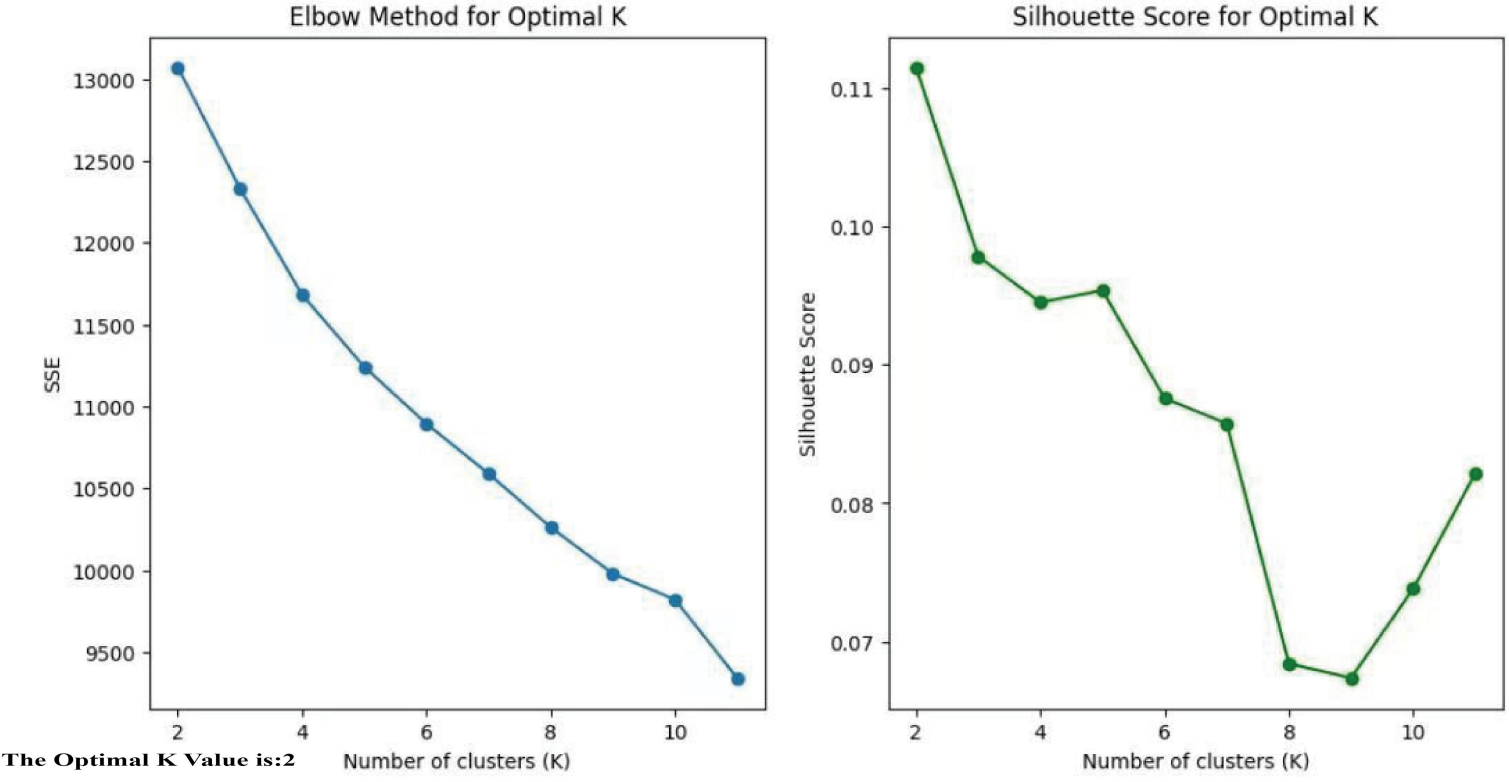
The optimal K.

By using the unsupervised clustering method, the optimal number of clusters that best explained the overall variance in the data was determined.(cluster group 1: n=262, cluster group 2: n=375). The clinical variables and myocardial ischemia characteristics were analyzed and compared across the two cluster subgroups. The analysis was based on Python 3.9.12 with the following libraries: scikit-learn 1.0.2 for implementing K-means clustering and calculating the Silhouette Coefficient, NumPy 1.21.5 for numerical computations, and Matplotlib 3.5.1 for visualizing the Elbow Method and Silhouette Score plots. The Jupyter Notebook environment was used for code development and execution (**Figure 3**).

### 2.5 Statistical analysis

Following cluster group identification, clinical information and myocardial ischemia characteristics were compared between the groups. Statistical analyses were conducted with SPSS software (version 25.0; IBM, Armonk, NY, USA). Categorical variables are presented as counts (%), while continuous variables are expressed as mean ± standard deviation. T-tests and chi-square tests were used to compare clinical and SPECT characteristics between cluster groups. A two-tailed P value < 0.05 was considered statistically significant.

## 3 Result

### 3.1 Study population

The study included a total of 637 individuals with T2DM,of whom 68.3% (435/637) were men, with an average age of 61.65 ± 14.05 years old. Unsupervised K-means clustering analysis was performed on 29 clinical parameters of 637 T2DM subjects. The silhouette coefficient for different values of K was calculated, and the K value with the highest silhouette coefficient was selected as the optimal number of clusters. The results revealed the existence of two clinical subtypes of T2DM with myocardial ischemia, which were classified into Cluster 1 (n=262) and Cluster 2 (n=375). **Table 1** presents the clinical characteristics of the participants in the two cluster groups.

**Table 1:** Baseline characteristics of the study cohort.

Variables	Cluster1 (n=262)	Cluster2 (n=375)	P value
Male(%)	177(67.6%)	258(68.8%)	0.740
Age (years old))	63.23±12.31	60.49±15.04	0.009
BMI (kg/m2)	25.22±4.03	25.46±4.43	0.469
Smoking history (%)	111(42.4%)	167(44.5%)	0.587
Alcohol (%)	74(28.2%)	82(21.9%)	0.065
Hypertension (%)	82(34.9%)	75(35.5%)	0.778
Systolic blood pressure (mmHg)	132.70±22.85	129.84±20.79	0.101
Diastolic blood pressure (mmHg)	77.30±14.71	77.81±15.32	0.666
Pulse (rate)	77.53±14.71	80.29±16.57	0.011
Diabetes duration(year)	10.27±8.77	5.51±7.61	<0.001
HbA1c (%)	7.69±1.76	6.35±1.68	<0.001
Fasting blood glucose(mmol/L)	8.29±7.26	6.44±2.62	<0.001
Cholesterol(mmol/L)	3.90±1.25	3.96±1.12	0.459
Triglyceride(mmol/L)	1.61±1.04	1.57±0.82	0.066
HDL-C(mmol/L)	2.51±1.02	2.52±0.83	0.178
LDL-C(mmol/L)	1.03±0.38	2.08±0.32	0.728
Serum uric acid (µmol/L)	390.56±130.98	406.78±135.24	0.066
Serum Creatinine(µmol/L)	115.42±106.18	106.38±102.70	0.009
Diabetes treatment (%)			
Oral			
Biguanides	64(24.4%)	71(18.9%)	0.095
Non-Sulfonylureas	7(2.7%)	14(3.7%)	0.997
α-Glucosidase inhibitor	61(23.3%)	46(12.3%)	<0.001
Sulfonylureas	21(8.0%)	15(0.4%)	<0.001
GLP-1 receptor agonist	2(0.8%)	26(6.9%)	0.076
DPP-4 inhibitor	11(4.2%)	21(5.6%)	0.713
SGLT-2 inhibitor	35(13.4%)	36(9.6%)	0.082
Stains	1(0.4%)	23(6.1%)	0.013
Without drugs	41(15.6%)	82(21.9%)	<0.001
Insulin	106(40.5%)	58(15.5%)	<0.001

Statins in Cluster 2 were likely prescribed at low-to-moderate intensity, which may partially explain the inconsistency with ischemia outcomes reported in high-dose statin clinical trials.Cluster 1 patients showed higher use of SGLT2 inhibitors and insulin, while Cluster 2 patients were more likely to receive GLP-1RA, DPP-4 inhibitors, and statins.

BMI, Body Mass Index; HbA1c, HbA1c, Glycated Hemoglobin; LDL-C, low-density lipoprotein cholesterol; HDL-C, High-density lipoprotein cholesterol; GLP-1,Glucagon-Like Peptide-1receptor agonist; DPP-4 inhibitor, Dipeptidyl Peptidase-4 inhibitor; SGLT-2 inhibitor, Sodium-Glucose Co-Transporter 2 inhibitor;

#### 3.1.1 Cluster group 1

In Cluster 1, the proportions of male patients (67.6%), smoking history (42.4%), and hypertension (34.9%) were lower than those in Cluster 2, and serum uric acid levels were significantly lower. Compared with Cluster 2, Cluster 1 participants had a higher prevalence of alcohol consumption history (28.2%*vs*21.9%) (Alcohol consumption history was defined as current drinking at the time of the study, past drinking, or both.)and a longer duration of diabetes (10.27 ± 8.77 years *vs*5.51 ± 7.61years). Regarding treatment patterns, patients in Cluster 1 were more likely to use metformin, α-glucosidase inhibitors, sulfonylureas, SGLT-2 inhibitors, and insulin, whereas a higher proportion of Cluster 2 patients were untreated or received GLP-1 receptor agonists and statins (**Table 1**).

#### 3.1.2 Cluster group 2

Cluster group 2 patients had a reduced period of diabetes(5.51 ± 7.61years) and had the lower Rate of patients involving alcohol consumption (21.9%).A larger fraction of patients in cluster group 2 had received diabetes treatment, like non-Sulfonylureas, Glucagon-Like Peptide-1(GLP-1) receptor agonist, Dipeptidyl Peptidase-4(DPP-4) inhibitor, Statins. Moreover, without drug treatment, Cluster 1 had 141 patients (15.6%), while Cluster 2 had 82 patients (21.9%), showing a significant difference (p < 0.001).

### 3.2 Connection between cluster identity and myocardial ischemia

**Figure 4:**
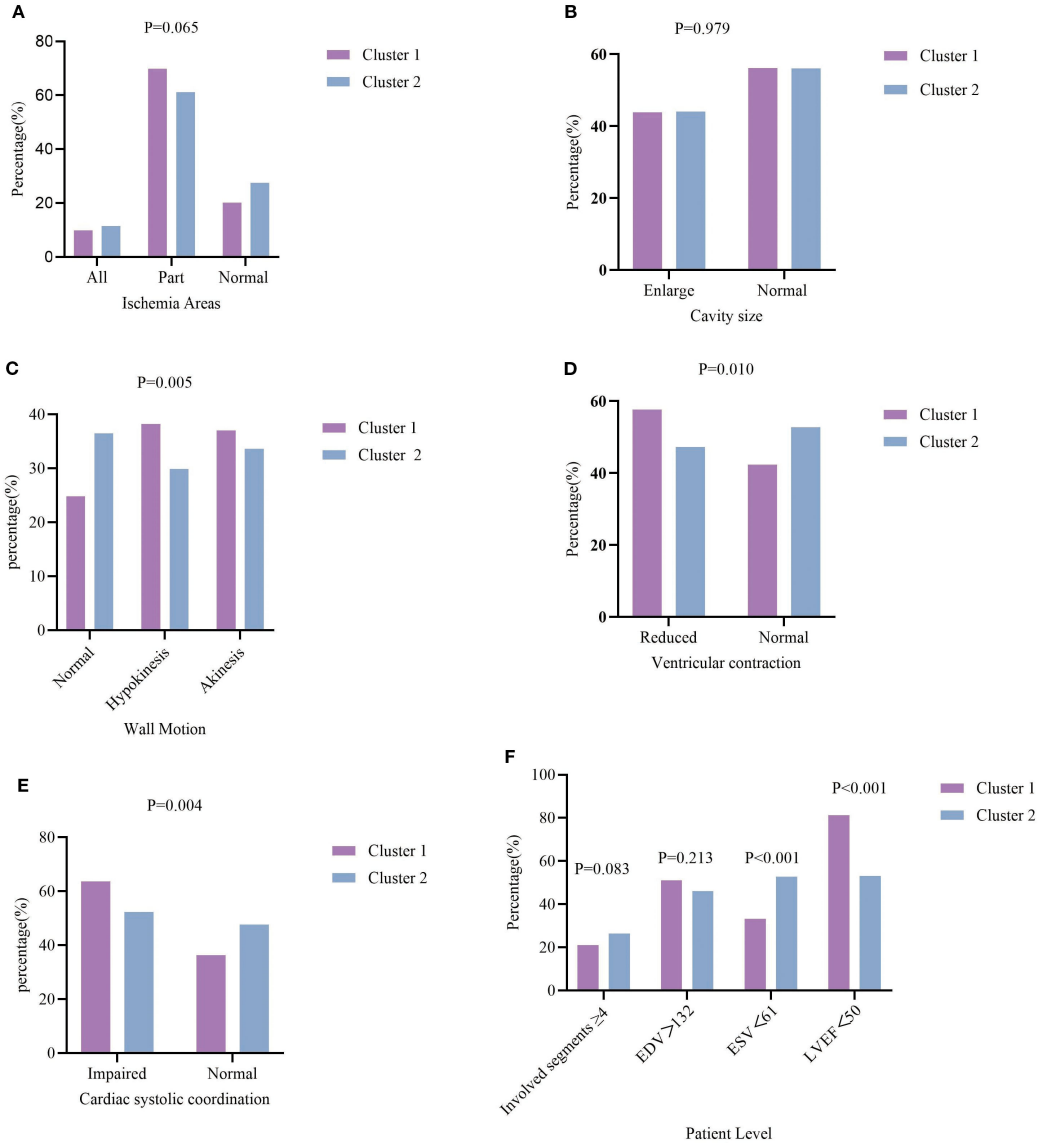
Characteristics of myocardial ischemia among the two clustering groups. **(A)** percentage of T2DM patients with different extent of ischemia; **(B)** percentage of Cavity size; **(C)** percentage of wall Motion; **(D)** percentage of ventricular contraction; **(E)** percentage of cardiac systolic coordination; **(F)** percentage of T2DM patients with involved segments ≥4, EDV, ESV, LVEF.

The characteristics of myocardial ischemia are compared across the two clustering groups in **Table 2** and **Figure 4**. In terms of Ischemia areas, cluster group 1 had the higher proportion of patients with incomplete ischemia (69.8%), Cluster group 2 had higher proportion of complete ischemia (11.5%) (**Figure 4A**). Wall motion in cluster group 1 tend to had higher proportion of Hypokinesis and Akinesis, while normal wall motion in cluster group 2 seems more common (**Figure 4C**). For Ventricular contraction, cluster group 1 had worse contraction (**Figure 4D**). In terms of Cardiac systolic coordination, group 1 had a worse proportion (**Figure 4E**). From the patient perspective, cluster group 1 exhibited a greater rate of abnormal ESV and LVEF (**Figure 4F**). No significant variations were noted in the involved segments, EDV, ischemia areas, and cavity size (**Figure 4B**) between the two cluster groups (all P values>0.05).

**Table 2:** Comparison of myocardial ischemia characteristics of two cluster groups in T2DM.

	Cluster(n=262)	Cluster2(n=375)	P value
Ischemia areas			0.065
All	26(9.9%)	43(11.5%)	
Part	183(69.8%)	229(61.1%)	
Normal	53(20.2%)	103(27.5%)	
Cavity size			0.979
Enlarge	115(43.9%)	165(44%)	
Normal	147(56.1%)	210(56%)	
Wall Motion			0.005
Normal	65(24.8%)	137(36.5%)	
Hypokinesis	100(38.2%)	112(29.9%)	
Akinesis	97(37.0%)	126(33.6%)	
Ventricular contraction			0.010
Reduced	151(57.6%)	177(47.2%)	
Normal	111(42.4%)	198(52.8%)	
Cardiac systolic coordination			0.004
Impaired	167(63.7%)	196(52.3%)	
Normal	95(36.3%)	179(47.7%)	
Patient Level			
Involved segments ≥4	55(21.0%)	99(26.4%)	0.083
Involved segments <4	207(79.0%)	276(73.6%)	
EDV>132	134(51.1%)	173(46.1%)	0.213
EDV<132	128(48.9%)	202(53.9%)	
ESV>61	175(66.8%)	177(47.2%)	<0.001
ESV<61	87(33.2%)	198(52.8%)	
LVEF<50	213(81.3%)	199(53.1%)	<0.001
LVEF>50	49(18.7%)	176(46.9%)	

## 4 Discussion

In this research, an unsupervised machine learning method was applied to classify T2DM patients into subgroups with different clinical profiles. Machine learning methods provide innovative approaches to integrate and analyze diverse omics data, facilitating disease-Specific biomarker discovery. These biomarkers provide the opportunity to enhance prognostic assessment accuracy, stratified healthcare, and the delivery of precision medicine (25, 26). This study showed that unsupervised learning techniques can be applied to analyze integrated healthcare data and enable the possibility of identify distinct T2DM patient categories with varying ischemia areas and degrees of co myocardial ischemia.

### 4.1 Unsupervised machine learning for processing clinical data

It is widely recognized that diabetic patients are at greater risk for more aggressive vascular disease, including diffuse coronary atherosclerosis, and exhibit a significantly increased occurrence of heart failure, myocardial infarction (MI), and cardiovascular mortality (27, 28). Diabetic patients have multitude of characteristic features, the interaction of these contributor on myocardial ischemia are attracting more focus. Preceding studies primarily aimed at the limited factors for myocardial ischemia (29–31). The target of unsupervised machine learning is to uncover clusters of patients with analogous combinations of features, free from biases introduced by clinical experts or information on future outcomes. As clinical data continue to grow rapidly, clustering methods may become increasingly valuable for analyzing the varied and multifaceted data available processing the diverse and heterogeneous data found in digital health data.

### 4.2 Correlation between myocardial ischemia areas and clusters

The clustering approach demonstrated the ability to not only differentiate T2DM patients with diverse clinical profiles but also to indirectly identify distinct subgroups exhibiting various types of myocardial ischemia. The findings revealed that cluster 2 had higher proportion of complete ischemia. This can be attributed to the evidence that cluster group 2 consisted of a larger number of males, exhibited more unhealthy habits such as nicotine use and alcohol consumption, and had elevated levels of LDL-C. Present smoking has been classified as a threat for myocardial ischemia (32). Mild alcohol consumption is known to be cardio protective compared with either heavy drinking or complete abstinence (33, 34). Treatment to reduce LDL cholesterol HDL-C levels is beneficial to improve ischemia (35). This result draws attention to the crucial role of effective management for T2DM patients exhibiting these risk factors for myocardial ischemia.

In line with these risk profiles, patients in Cluster 2 were more frequently prescribed GLP-1 receptor agonists and statins. GLP-1 receptor agonists have been reported to improve coronary microvascular function, while statins effectively reduce LDL-C concentrations. Nevertheless, in this cohort statin therapy was primarily administered at low-to-moderate intensities, which may have attenuated their cardioprotective effect. This limitation could partly explain the persistently higher prevalence of complete ischemia in Cluster 2, despite the seemingly more optimized pharmacological regimen.

### 4.3 Heart motion function in clusters

Group1 had a higher proportion of hypokinesis and akinesis wall motion, and ventricular contraction and cardiac systolic coordination also worse in group 1. This might be attributed to the fact that patients in Cluster 1 were older (63.23 ± 12.31 *vs*60.49 ± 15.04 years), had a longer duration of diabetes (10.27 ± 8.77*vs*5.51 ± 7.61 years), and exhibited poorer glycemic control, as reflected by higher HbA1c levels (7.69 ± 1.76*vs*6.35 ± 1.68).A previous study showed that wall motion is an independent predictor of ischemic heart (36, 37). In both cross-sectional and short-term longitudinal studies involving older adults, the status of glycemic dysregulation add to the risk of wall motion (38, 39). Additionally, inadequate control of diabetes mellitus (DM) was associated with subclinical left ventricular (LV) dysfunction (40). Traditionally, aging is regarded as a risk factor for myocardial ischemia. We infer from our data that longstanding glycemic abnormality produces a compounded harmful influence on LV wall motion.

Although SGLT2 inhibitors were more commonly prescribed in Cluster 1 as a cardioprotective strategy, the prevalence of reduced exercise tolerance and exercise incapacity remained high. This apparent inconsistency highlights that, despite the demonstrated efficacy of SGLT2 inhibitors in randomized controlled trials, real-world effectiveness may be attenuated due to suboptimal adherence, heterogeneity in therapeutic responsiveness, or patient-specific factors. These findings underscore the need for further evaluation of treatment strategies in routine clinical practice to mitigate LV dysfunction in high-risk T2DM populations.

### 4.4 Association of extent of ESV and LVEF with ischemia

Although some differences were observed in ventricular contraction, cardiac systolic coordination, ESV and LVEF, there was no statistically significant difference in the ischemic areas between the two cluster groups. A previous study demonstrated that T2DM increase the risk of death among patients with ischemic heart disease. Another studies showed that risk factors for myocardial ischemia, such as hypertension, hyperlipidemia, diabetes, smoking, obesity, age, gender, family history, can exacerbate the severity of myocardial ischemia through different mechanisms (41–43). These risk factors collectively contribute to the increased severity of myocardial ischemia. Therefore, early intervention and comprehensive management targeting these factors are crucial.

Beyond systolic function, diastolic impairment also represents a critical concern in T2DM. The disease promotes myocardial fibrosis and increases ventricular stiffness, thereby contributing to diastolic dysfunction—a hallmark mechanism of heart failure with preserved ejection fraction (HFpEF). In line with this, Cluster 1 patients demonstrated more pronounced abnormalities in left ventricular end-diastolic volume (EDV) and E/E′ ratio, indicating a heightened susceptibility to diastolic dysfunction. Importantly, these patients also exhibited higher mean systolic blood pressure, longer disease duration, and poorer glycemic control, whereas Cluster 2 patients presented with slightly lower diastolic pressure. Consequently, a wider pulse pressure was evident in Cluster 1. Given that both hypertension and diabetes are key risk factors for HFpEF, these pathophysiological distinctions provide additional mechanistic support for the clustering results.

### 4.5 Clinical implications of unsupervised clustering

The present study demonstrates the feasibility of applying unsupervised clustering to classify T2DM patients with myocardial ischemia and underscores its potential clinical relevance. By identifying subgroups with distinct ischemic and metabolic features, clustering provides an evidence-based approach for personalized decision-making, enabling physicians to tailor therapies to specific risk profiles. Its integration of SPECT-derived functional parameters further highlights potential for early ischemia detection, thereby improving diagnostic accuracy and guiding timely interventions in high-risk populations. Beyond diagnosis, clustering may assist in predicting disease progression and stratifying cardiovascular risk, offering a valuable tool for long-term management and proactive prevention. Overall, these findings suggest that unsupervised learning could complement conventional risk assessment and foster a more precise and individualized model of care for patients with T2DM.

## 5 Limitations

This study has several limitations. First, given that it is a monocentric study, Systematic bias in selection is unavoidable, and future multi-center studies are required to confirm these findings. Second, since it was a retrospective analysis, ongoing data were not available. The gradual onset and progression of myocardial ischemia in T2DM patients requires in-depth study. However, SPECT, a noninvasive examination, is one of the most common imaging modalities used for assessment of ischemia (44).

## 6 Conclusions

This study demonstrates that unsupervised clustering methods can effectively analyze heterogeneous clinical and imaging data from T2DM patients with myocardial ischemia. By revealing distinct patient subgroups, this approach provides a novel pathway for stratifying individuals with varying ischemic burdens. These findings highlight the methodological value of unsupervised learning for integrating complex healthcare data and suggest promising directions for future research aimed at refining risk stratification and improving outcomes in T2DM populations.

## Data Availability

The data analyzed in this study is subject to the following licenses/restrictions: Medical data, involving ethics and privacy. Requests to access these datasets should be directed to BL, liubing@sxmu.edu.cn.
